# IL-27 Derived From Macrophages Facilitates IL-15 Production and T Cell Maintenance Following Allergic Hypersensitivity Responses

**DOI:** 10.3389/fimmu.2021.713304

**Published:** 2021-09-30

**Authors:** Jutamas Suwanpradid, Min Jin Lee, Peter Hoang, Jeffery Kwock, Lauren P. Floyd, Jeffrey S. Smith, Zhinan Yin, Amber R. Atwater, Sudarshan Rajagopal, Ross M. Kedl, David L. Corcoran, Jennifer Y. Zhang, Amanda S. MacLeod

**Affiliations:** ^1^ Department of Dermatology, Duke University, Durham, NC, United States; ^2^ Department of Molecular Genetics and Microbiology, Duke University, Durham, NC, United States; ^3^ Department of Biochemistry, Duke University, Durham, NC, United States; ^4^ Zhuhai Institute of Translational Medicine Zhuhai People’s Hospital Affiliated with Jinan University, Jinan University, Zhuhai, China; ^5^ The Biomedical Translational Research Institute, Faculty of Medical Science, Jinan University, Guangzhou, China; ^6^ Department of Medicine, Duke University, Durham, NC, United States; ^7^ Department of Immunology and Microbiology, University of Colorado Anschutz School of Medicine, Aurora, CO, United States; ^8^ Center for Genomic and Computational Biology, Duke University, Durham, NC, United States; ^9^ Department of Pathology, Duke University, Durham, NC, United States; ^10^ Department of Immunology, Duke University, Durham, NC, United States

**Keywords:** contact hypersensitivity, human allergic contact dermatitis, IL-27, IL-15, dermal leukocyte cluster, BCL2, CD172a, STAT1

## Abstract

Crosstalk between T cells, dendritic cells, and macrophages in temporal leukocyte clusters within barrier tissues provides a new concept for T cell activation in the skin. Activated T cells from these leukocyte clusters play critical roles in the efferent phase of allergic contact hypersensitivity (CHS). However, the cytokines driving maintenance and survival of pathogenic T cells during and following CHS remain mostly unknown. Upon epicutaneous allergen challenge, we here report that macrophages produce IL-27 which then induces IL-15 production from epidermal keratinocytes and dermal myeloid cells within leukocyte clusters. In agreement with the known role of IL-15 as a T cell survival factor and growth cytokine, this signaling axis enhances BCL2 and survival of skin T cells. Genetic depletion or pharmacological blockade of IL-27 in CHS mice leads to abrogated epidermal IL-15 production resulting in a decrease in BCL2 expression in T cells and a decline in dermal CD8^+^ T cells and T cell cluster numbers. These findings suggest that the IL-27 pathway is an important cytokine for regulating cutaneous T cell immunity.

## Introduction

Delayed-type IV hypersensitivity responses against epicutaneous contact allergens manifest clinically as allergic contact dermatitis (ACD) in humans and as allergic contact hypersensitivity (CHS) in mice. ACD is one of the most common skin inflammatory diseases in the United States and remains the primary cause of occupational skin disease (1–3). Cutaneous contact allergens (also referred to as haptens when they bind larger protein carriers to elicit allergy responses) are presented by dendritic cells (DCs) to naïve T cells, which then become allergen-specific T cells. These T cells are required for initiating a robust skin inflammatory response upon re-exposure of the skin to the same allergen. As the skin inflammation resolves, effector T cells (CD8^+^ T cells in particular) undergo apoptotic cell death or develop into memory T cells, including tissue-resident memory T cells (T_RM_) (4, 5). Concomitant with the frequency of antigen (Ag)/allergen exposure, T_RM_ cells of the skin persist and accumulate around the hair follicle epithelium to develop immunity against re-exposure to the same Ag (6–8). Such T_RM_ cells have been described to be also responsible for the severity of the skin inflammatory response during CHS (4). This knowledge is in agreement with the clinical observation that patients with ACD frequently suffer recurrence and worsening of skin contact dermatitis after being repetitively exposed to the same allergen over time.

IL-27 is a new member of the IL-12 family of heterodimeric cytokines and is comprised of the IL-27p28 and Epstein-Barr virus-induced gene 3 (EBI3) subunits (9). It is produced predominantly by myeloid cells and signals through a receptor comprised of WSX-1/TCCR (IL-27RA) and gp130 subunits, expressed by both innate and adaptive immune cells. IL-27 has been linked to a wide array of cellular and molecular immune responses and inflammatory diseases (10–26) and has been detected in acutely and chronically inflamed eczematous skin, including psoriasis and atopic dermatitis (21, 22, 24). IL-27 has been shown to regulate T cell differentiation (10–26), and it can stimulate keratinocytes to release Th1-attracting chemokines such as CXCL10, thereby maintaining inflammatory cell recruitment (24). Global deletion of *Il-27ra* or *Ebi3* in mice has been reported to result in various outcomes on inflammatory disorders (17, 23). Similarly, supplementation of IL-27 led to differential inflammatory responses *in vivo* and *in vitro* that can likely be attributed to tissue context-specific effects (17, 25, 27).

In addition to T cells, macrophages (MACs) and DCs are key immune cells in CHS and have been recently identified by us and others in the ACD-associated dermal leukocyte clusters (28, 29). These dermal leukocyte clusters, even though they resemble some morphological similarities to tertiary lymphoid structures (TLS), are currently not known to provide a niche for ectopic lymphoneogenesis, a hallmark of TLS (30). Instead, these transient dermal leukocytes often surround or are in close proximity to small blood or lymphatic venules and appear to correlate with severity of the skin inflammatory response and blister formation in human ACD patients.

Here, we report that IL-27 is produced by a CD172a^+^ MAC subset following epicutaneous allergen exposure in humans and mice. Using both Il-27p28fl/fl;LysMCre mice and pharmacological inhibition of IL-27, we demonstrate that inhibition of IL-27 abrogated epidermal IL-15 production, leading to a decrease in BCL2 expression and survival in skin T cells following CHS.

## Methods

### Human Subjects, Study Approval, and Skin Biopsy Samples

All studies involving human subjects were approved by the Institutional Review Board of Duke University Health System, and such protocols allowed the use of de-identified specimens for future research. Study participation inclusion was offered to patients undergoing patch testing in a specialty contact dermatitis clinic. Inclusion criteria were ≥18 years of age and completion of patch testing. Exclusion criteria were pregnancy, topical corticosteroids at patch site, oral corticosteroids, systemic immunosuppressants, phototherapy, known bleeding disorders, and allergy to lidocaine or epinephrine (31). Skin biopsies were obtained from male and female volunteers undergoing patch-testing and as part of the above-mentioned approved protocol. Patches containing test allergens were applied to study participants on day 1, removed on day 3, and read at 96 to 120 hours. If a study participant had a positive patch test, then a 4-millimeter punch biopsy at the test site (positive patch test) and a 4-millimeter punch biopsy at a negative site (control) were obtained from normal regions of skin nearby.

### Skin Explant T Cells Preparation and Culture

Human skin specimens were collected from healthy patients undergoing plastic surgery at Duke University Medical Center and used anonymously. All human samples for this study were obtained according to the protocols approved by the Institutional Review Board at Duke University. Samples of normal human skin obtained were cultured in 24-well plates. The human skin samples were incubated in skin explant media modified from Clark et al. (32) (DMEM; 10% FBS; 0.1 mM non- essential amino acids (Thermo Fisher Scientific, Waltham, MA); 1 mM sodium pyruvate; 2 mM L-Glutamine; 1% Pen/Strep (Thermo Fisher Scientific); IL-2 (5 unit/ml, PromoCell, Heidelberg, Germany); and IL-15 (7.5 ng/ml, Tonbo Biosciences, San Diego, CA). For other experiments, cells were then cultured in skin explant media without IL-2 and IL-15 for 24 hours before being collected. Cells that migrated into the culture media were harvested and utilized for further FACS sorting. FACS-sorted T cells were treated with recombinant 2 nM IL-15 or 3.1 nM IL-27 (BioLegend, San Diego, CA) or vehicle control for 24 hours and then collected for flow analysis.

### Human Keratinocytes

Normal human epidermal keratinocytes (NHEKs) were purchased from Thermo Fisher Scientific and maintained for up to 6 passages in T-75 flasks or used earlier. Cells were grown in serum-free EpiLife cell culture medium with EpiLife Defined Growth Supplement containing 0.06 mM Ca^2+^ (Gibco, Waltham, MA) or Keratinocyte serum-free-media (KSFM) with supplements provided by manufacturer (Gibco) and additional 0.06mM Ca^2+^. NHEKs were grown to approximately 75-80% confluence. For experiments, cells between passage 3-6 were plated at approximately 200,000 cells/well in 6-well plates and 75,000 cells/chamber in 2-chamber slides, respectively (LabTek, Bloomington, IN). For some experiments, IL-27 was used at a concentration of 100 ng/mL; IFN-α was used at a concentration of 50 U/mL (BioLegend). The cells were collected for quantitative RT-PCR or immunofluorescence at various time points.

### Hapten Stimulation of Human THP-1 Cells

Human monocytic THP-1 cells were purchased from ATCC and maintained for up to 15 passages in T-75 flasks. Cells were grown in DMEM (Corning Incorporated, Corning, NY) with 10% fetal bovine serum (FBS, Sigma-Aldrich, St. Louis, MO). For experiments, cells were plated at 750,000 cells/well in 6-well plates and treated with NiCl_2_ (100 µM, Sigma-Aldrich), 2,4-dinitrobenzene sulfonic acid sodium salt (DNBS) (0.05%, Sigma-Aldrich), or vehicle for multiple time points before harvest in TRIzol (Thermo Fisher Scientific). Cells were treated with monensin (Thermo Fisher Scientific) and brefeldin* *A (Sigma-Aldrich) for 3 hours prior to collection for cell immunofluorescence staining.

### Mice and Study Approval

All animal studies were approved by the Duke University Institutional Animal Care and Use Committee under protocols A175-14-07, A156-17-06, and A107-20-05. C57BL6/J (The Jackson Laboratory, Stock #000664, Bar Harbor, ME), Il-27p28fl/fl;LysMCre^+/^ *^-^*, Il-27p28^EGFP^ mice and their littermates or Il-27p28fl/fl mice were used for control. Il-27p28^EGFP^ mice were kindly generated (14) and provided by Dr. Ross M. Kedl. Il-27p28fl/fl mice (33) were kindly provided by Drs. Zhinan Yin (Biomedical Translational Research Institute, Jinan University) and Li Fan Lu (University of California San Diego). These mice were bred with LysMCre mice (The Jackson Laboratory, Stock #004781) to generate Il-27p28fl/fl;LysMCre^+/^ *^-^* in our laboratory. Mice were maintained under regulated conditions with food and water *ad libitum* in the pathogenic-free facility at Duke University.

### CHS Mouse Model

For the allergic CHS model, mice were sensitized *via* topical application of 0.5% (v/v) 1-Fluoro-2,4-dinitrobenzene (DNFB) (Sigma-Aldrich) in 4:1 acetone/olive oil on their shaved back (50 µL) and were challenged 4 or 5 days later with 0.2% DNFB or vehicle control (5 µL on the dorsal and 5 µL on the ventral side of the ear). Ear thickness was measured using an engineer’s micrometer (Mitutoyo, Kawasaki, Kanagawa, Japan). The mice received neutralizing IL-27 antibody (nIL-27p28AB) (R&D Systems, Minneapolis, MN), IL-15 complex (cpx), or their respective IgG control (Ctrl). To make approximately 1 µg of the IL-15 cpx, 1 µg of IL-15 (PeproTech, Rocky Hill, NJ) and 4.5 µg of IL-15 Rα (R&D Systems) were incubated for 30 minutes at 37°C (34). Each mouse received 1.2 µg of IL-15 of the cpx.

### Repetitive DNFB Mouse Model

Mice were sensitized *via* topical application of 0.1% (v/v) DNFB (Sigma-Aldrich) in 4:1 acetone/olive oil on their shaved back (50 µL), 5 µL on the dorsal, and 5 µL on the ventral side of the ear. Then, the mice were challenged for a total of 3 times to generate stable T cell clusters. A single dose (12 µg) of the nIL-27p28AB (R&D Systems), anti-CD122 (66 µg, BioXcell, Lebanon, NH), or goat IgG (12 µg, R&D Systems), rat IgG (66 µg, BioXcell) was injected intradermally (i.d.) on mice ears. The ears were collected for gene and flow analysis at various time points after the administration. The mouse back was injected with nIL-27p28AB or Goat IgG (6 µg, daily i.d.) for a total of 3 times, and the back skin was harvested after 6 hours for immunofluorescence analysis.

### Generation of Bone Marrow-Derived DCs (BMDC) and Bone Marrow-Derived MACs (BMDM)

Bone marrow cells were stimulated with GM-CSF (20 ng/ml, BioLegend) and IL-4 (50 ng/ml; Tonbo Biosciences) to induce bone marrow–derived DCs (BMDC). Bone marrow-derived MACs (BMDM) were generated *via* culture with M-CSF (20 ng/ml; Sigma-Aldrich). On day 5, cells were used for experiments or collected for gene analysis.

### Epidermal and Dermal Ear Sheet Separation

Murine ear tissues were separated and floated on Trypsin GNK (0.3% Trypsin, 0.1% glucose, 14.8 mM NaCl, 5.3 mM KCl; Sigma Aldrich) for 15 min at 37°C. The epidermis and dermis were separated and collected in TRIzol reagent.

### Small Interfering RNA (siRNA) Knockdown

siRNA constructs were obtained from OriGene (Rockville, MD) or Dharmacon (Lafayette, CO). GenMute™ siRNA Transfection Reagent kit was used for siRNA transfection (SignaGen Laboratories, Frederick, MD). Cells were plated 1 day prior to transfection and media was changed to serum-free media 2 hours prior to transfection. siRNA constructs were resuspended in 1X transfection buffer at a concentration of 0.02 nmol/µL. 1.7 µL of each construct was added to a 200 µL master mix that contained both 1X transfection buffer and transfection reagent at volumes indicated by the manufacturer. siRNA master mixes were allowed to incubate at room temperature for 15-30 minutes before being added dropwise to wells (100µL per well). 5 hours after siRNA master mixes were added, media was changed to serum-containing media, and cells were stimulated with the indicated cytokines as described above. Scramble siRNA was used as control (SR30004, OriGene).

### Quantitative RT-PCR

Total RNA was isolated from cells and tissue using TRIzol. RNA was reverse transcribed using the iScript cDNA synthesis kit (Bio-Rad, Hercules, CA), and the resulting cDNA was amplified using the Fast Start Universal SYBR Green Master Mix (Thermo Fisher Scientific) or qPCRBIO SyGreen Blue Mix Hi-ROX (PCR Biosystems, London, England). PCR was performed with primers as shown in **Supplementary Materials**. Fold induction of gene expression was normalized to the house keeping gene glyceraldehyde-3-phosphate dehydrogenase (GAPDH) and calculated using the 2(−Δ−ΔCt) method (35).

### Immunofluorescence

Sections of frozen specimens (either human or mouse) and cells were incubated overnight at 4°C with primary ABs anti-human or anti-mouse. Mouse IgG1 isotype control (MOPC-21) (Tonbo Biosciences), Goat IgG isotype control (R&D Systems), Sheep IgG isotype control (R&D Systems), Rabbit isotype control (Southern Biotech, Birmingham, AL), anti-human CD14 (61D3, Tonbo Biosciences), anti-human iNOS (polyclonal, Thermo Fisher Scientific), anti-human CD8 (MCD8, Santa Cruz Biotechnology, Dallas, TX), and IL27R (polyclonal, R&D Systems), anti-human IL-27 (polyclonal, R&D Systems), anti-human CD86 (IT2.2, Biolegend), anti-human CD3 (SP7, Abcam, Cambridge, England), anti-human CD47 (polyclonal, R&D Systems), anti-human SIRP alpha (CD172a) (OTI7B3, Origene), anti-human IL-15 (polyclonal, R&D systems), anti-human BCL2 (clone 100, BioLegend), anti-mouse CD3 (17A2, Tonbo Biosciences), and anti-mouse CD8 (YTS 105.18, Novus Biologicals, Littleton, CO) followed by reaction with Cy3, Alexa Fluor 555, Alexa Fluor 647, Alexa Fluor 488, or FITC-conjugated secondary antibodies (Thermo Fisher Scientific). Nuclei were counterstained with Hoechst 33342 (Thermo Fisher Scientific), washed in PBS, and mounted with Anti-fade mounting media (Thermo Fisher Scientific).

### Flow Cytometry and FACS

Antibodies and appropriate IgG controls were conjugated to FITC, Alexa Fluor 488, PE, PeCy5, PerCPCy5.5, PeCy7, AmCyan, Brilliant Violet 421, Pacific Blue, eFluor 450, allophycocyanin, Vio770, Brilliant Violet 510, Brilliant Violet 650, Brilliant Violet 711, Alexa Fluor 594, Alexa Fluor 647, Alexa Fluor 700, PE Texas red, BUV 737, BUV 395, Brilliant Violet 786, Brilliant Violet 510, eFluor 780, and allophycocyanin-Cy7. Antibodies used in the study include anti-human CD3 (UCHT1, Tonbo Biosciences), anti-human BCL2 (100, BioLegend), anti-human BCL-XL (H-5, Santa Cruz Biotechnology), anti-human CD45RO (UCHL1, BioLegend), anti-human/mouse CD11b (M1/70, Tonbo Biosciences), anti-mouse XCR1 (ZET, BioLegend), anti-mouse Ly6C (HK1.4, BioLegend), anti-mouse CD3 (17A2, BioLegend), anti-mouse CD3e (145-2C11, Tonbo Biosciences), anti-mouse NK1.1 (CD161) (PK136, Tonbo Biosciences), anti-human/mouse B220 (CD45R) (RA3-6B2, Tonbo Biosciences), anti-mouse CD64 (X54-5/7.1, BioLegend), anti-mouse Ly6G (RB6-8C5, BioLegend), anti-mouse Ly6G (1A8, BioLegend), anti-mouse CD90.2 (Thy-1.2) (53-2.1, BioLegend), anti-human/mouse CD44 (IM7, BioLegend), anti-mouse CD24 (M1/69, BioLegend), anti-mouse CD45 (30-F11, BioLegend), anti-mouse CCR2 (475301, R&D Systems), anti-mouse CD172a (P84, BD Biosciences), anti-GFP (polyclonal, Thermo Fisher Scientific), anti-mouse CD8a (53-6.7, Tonbo Biosciences), and anti-mouse BCL2 (BCL/10C4, BioLegend). Cells were captured with DiVa 5.0 software on a digital LSRII and Fortessa analyzed with FlowJo software (FlowJo LLC, Ashland, OR).

To produce mice skin cell suspensions, skin pieces were manually disrupted using scissors and were processed using Trypsin GNK (0.3% trypsin, 0.1% glucose, 14.8 mM NaCl, 5.3 mM KCl) (Sigma-Aldrich), 0.1% DNase (Sigma-Aldrich), Dispase II (1.33 mg/mL, Worthington, Lakewood, NJ), Collagenase type II (2 mg/mL, Thermo Fisher Scientific) at a 6:1:1:2 ratio for 45 minutes at 37°C with intermittent vigorous shaking for single-cell isolation.

For sorting mouse neutrophils, mouse bone marrow was purified by FACS sorting for CD45^+^Gr-1^+^ cells. The purity of sorted cell populations was above 90%.

For human T cell studies, skin cells were purified by FACS sorting for CD45^+^CD3^+^ cells. The purity of sorted cell populations was above 90%.

### Analysis of Microarray Gene Expression Data

We obtained the raw microarray gene expression data produced by Pedersen et al. (36) from the Gene Expression Omnibus (accession number GSE6281) (37). The data were processed using the *affy* Bioconductor (38, 39) package from the R statistical programming environment. Robust Multiarray Average normalization was applied to the data to eliminate systematic differences across the dataset. The data was then filtered down to the 3 patients that had a positive reaction to the allergen and had data for both the 0 hr and 96 hours time points. A mixed-effect model with a moderated test statistic was run using the *limma* package (40). Probe sets were considered differentially expressed if they had a p-value ≤ 0.05 and at least a 50% increase or decrease in expression between the two time points.

### Venn Diagram

We identified the set of genes that had a p-value ≤ 0.05 and at least a 50% increase in expression in each of the three datasets. For the NHEK and THP1 datasets (accession number GSE143228) (27), the 50% increase in expression was in the stimulated versus unstimulated samples. For the ACD samples, the increase was in the 96 hours post-stimulation relative to the 0 hr sample.

### Software for Data Visualization and Analysis

The Pathway Commons website is an open-source database software representing physical interactions involving proteins, DNA, RNA, small molecules, and complexes (41). The data can be found in http://www.pathwaycommons.org/pcviz/#neighborhood/Il-27.

### Quantification and Statistical Analysis

For comparisons between multiple groups, the overall differences were analyzed by ANOVA with Bonferroni multiple comparison and least significance difference tests. For comparisons between two groups, two-tailed unpaired Student’s t*-*tests or paired Student’s t*-*tests (for same patients) were used. GraphPad Prism software (version 7, 8, and 9, San Diego, CA) was used for statistical analyses.

## Results

### Contact Allergen Exposure Upregulates IL-27 in Macrophages

**Figure 1:**
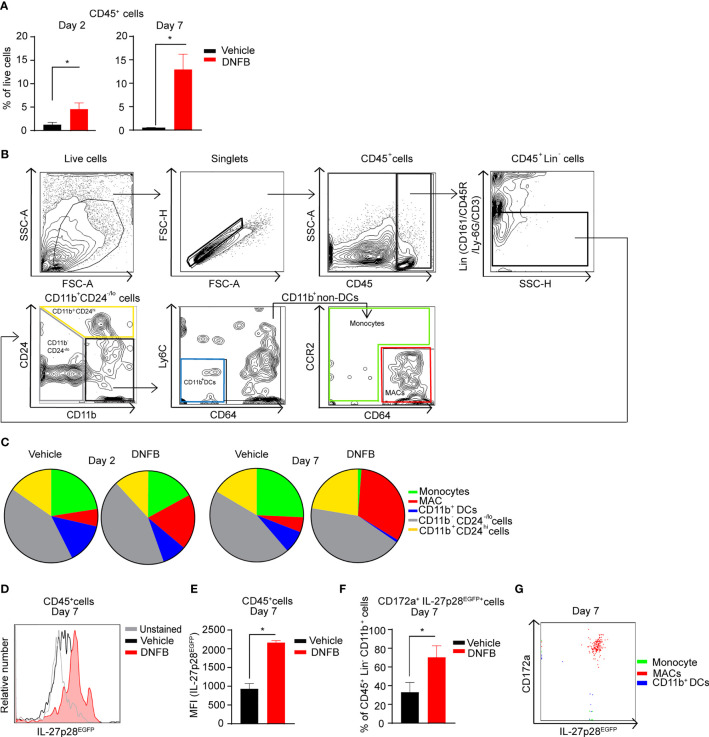
Exposure to allergen upregulates IL-27 in murine MACs during CHS. **(A)** Quantitative analysis of CD45^+^ hematopoietic cell frequency (in living cells) in DNFB-treated and vehicle-treated ear skin of the CHS mice (2 and 7 days post-DNFB elicitation). The data are represented as mean ± standard error of the mean (SEM) from at least 4 mice per group, *p < 0.05 (unpaired Student’s t test). **(B)** Gating strategies for skin myeloid cell population identification in the CHS mouse model. Single-cell suspensions of mouse ear skin treated were prepared. After excluding dead cells, as well as Lin^+^; including T cells, NK cells, B cells, and granulocytes, the remaining CD45^+^ cells were analyzed for expression of CD24 and CD11b. CD11b^+^CD24^-/lo^ cells were further analyzed for Ly-6C, CD64, and CCR2 expression. The CD11b^+^non-DCs fraction was separated into monocyte and macrophages (MACs) populations, respectively. **(C)** Pie charts summarizing immune cell distribution (gated on CD45^+^Lin^-^ cells) from mouse ear skin at 2 and 7 days post-DNFB elicitation. The data represent the mean of at least 4 mice per treatment group. **(D)** Histogram from representative flow cytometry analysis for IL-27p28 of vehicle-treated and DNFB-treated ears gated on CD45^+^ cells. Data shown are representative of at least 4 mice per group. **(E)** Data presented shows median fluorescence intensity (MFI) of IL-27p28 in the CD45^+^ population from ll-27p28^EGFP^ mice at 7 days post DNFB-elicitation versus vehicle controls from at least 4 mice per group and are depicted as mean ± SEM, *p < 0.05 (unpaired Student’s t test). **(F)** Quantitative analysis of CD172a^+^IL-27p28^EGFP+^ cell frequency of CD45^+^Lin^-^ CD11b^+^ cells in DNFB-treated and vehicle-treated ear skin. At least 4 mice per group and summarized as mean ± SEM, *p < 0.05 (unpaired Student’s t test). **(G)** Representative flow cytometric overlay dot plots of gated monocytes, MACs, and CD11b^+^ DCs at 7 days post-DNFB elicitation on mouse ear skin demonstrating CD172a and IL-27p28 expression from at least 4 mice per group.

First, we sought to characterize myeloid populations and identify the cell population that produces IL-27 using Il-27p28^EGFP^ mice in an allergic contact hypersensitivity (CHS) mouse model. As expected, using flow cytometry analysis, we first confirmed a significant increase in CD45^+^ hematopoietic cells infiltrating 1-Fluoro-2,4-dinitrobenzene (DNFB)-treated mouse ears at 2 and 7 days post-DNFB elicitation (**Figure 1A**). Using a more in-depth gating strategy adapted from Tamoutounour et al. (42), we analyzed DNFB-treated ears at 2 and 7 days post-DNFB elicitation and compared them to vehicle controls. A lineage (Lin) channel for CD161, CD45R, Ly6G, and CD3 was used to exclude natural killer (NK) cells, T cells, and granulocytes and allowed identification of distinct myeloid cell subsets based on CD24, CD11b, Ly6C, CCR2 and CD64 expression (**Figure 1B**). This analysis revealed a continuous expansion of macrophages (MAC) (CD45^+^Lin^-^CD11b^+^CD24^-/lo^CD64^hi^CCR2^-/lo^) at day 2 through day 7 post-DNFB. This expansion of MACs appeared inversely associated with a decrease in frequency of monocytes (CD45^+^Lin^-^CD11b^+^CD24^-/lo^) and CD11b^+^ dendritic cells (DCs) populations (CD45^+^Lin^-^CD11b^+^CD24^-/lo^CD64^-^Ly-6C^-^) (**Figure 1C**). On day 7 post-DNFB elicited ear skin, CD45^+^ hematopoietic cells up-regulated IL-27p28 (**Figures 1D, E**). Further analysis of the CD45^+^Lin^-^CD11b^+^ myeloid cell population revealed that the frequency of cells expressing IL-27p28 and myeloid inhibitory immune-receptor CD172a, also known as SIRPα, significantly increased (**Figure 1F**). These CD172a^+^ IL-27p28 co-expressing cells were comprised within the MAC subset (**Figure 1G**). No significant alteration of IL-27p28 expression was observed upon DNFB exposure in CD45^+^Lin^-^CD11b^-^ cells (comprised of CD11b^-^DCs abundantly expressing XCR1, not CD172a) (**Figure S1**).

**Figure 2:**
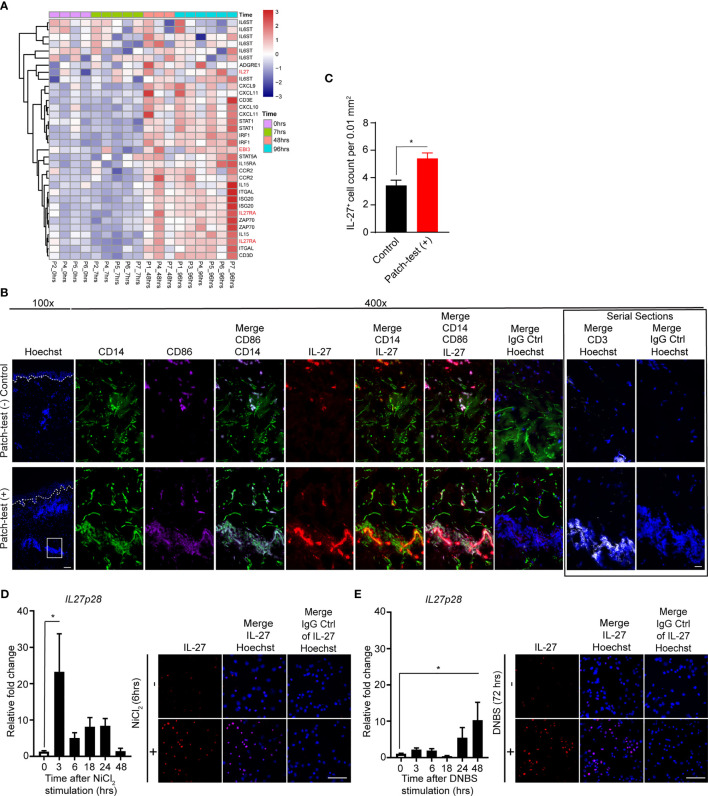
Myeloid cells produce IL-27 upon allergen exposure. **(A)** Heat map showing gene expression patterns of patch-tested skin from allergic contact dermatitis patients (GSE number: 6281). Samples for gene expression analysis were collected from positive patch-test reactions to nickel at 7, 48, and 96 hours post-elicitation as well as the 0h control. Samples and genes are clustered using correlation distance with complete linkage. **(B)** Representative immunofluorescence staining of IL-27 (red), CD14 (green), CD86 (purple), CD3 (white) and Hoechst (blue) in human donor-matched patch-test negative control and patch-test (+) ACD skin. Data are representative of 3 patient samples per stained condition. Original magnification x100 (left) and original magnification x400 (right) with scale bars 100 µm, and 20 µm, respectively. White dashed lines mark the epidermal-dermal junction. **(C)** Analysis depicting total numbers of dermal IL-27^+^ cells in donor-matched patch-test negative control and patch-test (+) ACD samples. Data are expressed as mean ± SEM from at least three separate microscopic fields from 3 patients, *p < 0.05 (unpaired Student’s t test). **(D, E)** Quantitative PCR and representative immunofluorescence staining of IL-27 of THP-1 cells treated with **(D)** nickel chloride (NiCl2, 100µM) and **(E)** dinitrobenzene sulfonic acid (DNBS, 0.05%) at various time points. Data are summarized as mean ± SEM from at least 3 biological replicates, *p < 0.05 (ANOVA test with Bonferroni correction).

We next screened previously published microarray data from skin samples of human sensitized allergic contact dermatitis (ACD) patients undergoing patch-testing (**Figure 2A**) (36). Clinically positive patch-test results at 48 and 96 hours post-elicitation showed induction of *IL27Ra, EBI3*, and *IL27p28 (IL-27)* (**Figure 2A**). Furthermore, a significant increase in IL-27^+^ cells was found within patch-test positive ACD skin lesions (**Figures 2B, C**). These cells were co-localized with CD14^+^CD86^+^ cells, likely representing monocyte-derived MACs or DCs, at 96 to 120 hours following patch test application relative to the donor-matched patch-test negative control (**Figures 2B, C**). IL-27^+^ cells were localized within the dermal leukocyte cell clusters (**Figure 2B**) and IL-27RA expression was found in both the epidermal and dermal compartments of patient samples (**Figure S2**). To determine whether allergens can directly induce IL-27 production in MAC, we next treated the human monocytic cell line, THP-1, with two common relevant haptens, NiCl_2_ and 2,4-dinitrobenzene sulfonic acid sodium salt (DNBS). Notably, these stimulations resulted in the upregulation of IL27p28 gene and protein expression (**Figures 2D, E**). Together, these results suggested that epicutaneous contact allergens lead to increase in IL-27 production in human and murine MACs.

**Figure 3:**
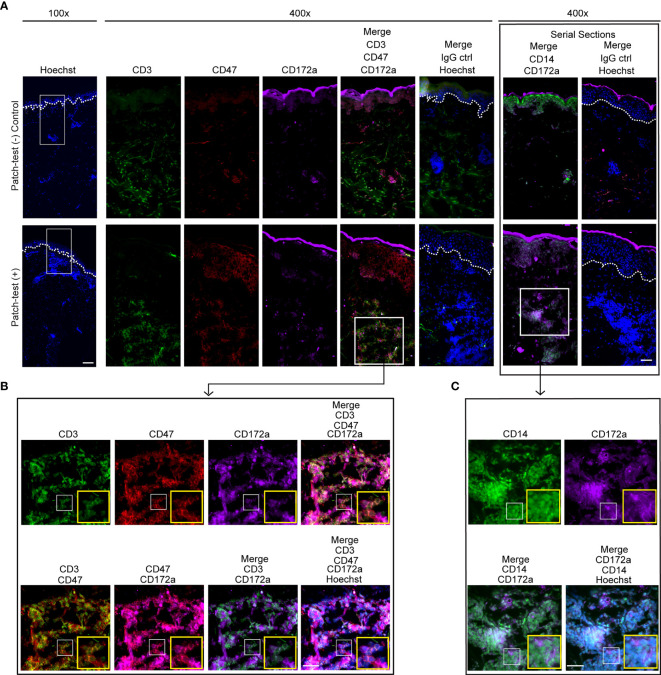
CD3, CD47, and CD172a expression in human ACD clusters. **(A–C)** Immunofluorescence staining of CD3 (green), CD47 (red), CD172a (purple), CD14 [green, a serial slide section with the staining of CD172a (purple)], and Hoechst (blue) in human donor-matched patch-test negative control and patch-test (+) ACD skin. White dashed lines mark the epidermal-dermal junction. Data are representative of 3 patient samples per tested condition. **(A)** Scale bars are 200 µm (left) and 100 µm (right). **(B, C)** Scale bars are 20 µm.

Dermal leukocyte clusters play an essential role in modulating CHS responses and are recognized as the hallmark of ACD disease (28, 29, 43). Concurrent with our murine CHS data (**Figure 1**), CD14 and CD172a positive cells were found adjacent to T cells in patch-test positive patient samples (**Figures 3A–C**). Skin T cells in these patient samples were comprised of CD47^+^ and CD47^-^ cells. CD47 is a membrane protein that interacts with CD172a and elicits “do not eat me” signals to phagocytic cells (44–46).

### Myeloid Cell-Derived IL-27p28 Is Essential to the CHS Response

To better delineate the role of IL-27 in myeloid cells, we next investigated the functional consequences of *Il-27p28* deletion in myeloid cells using Il-27p28fl/fl;LysMCre mice. Successful depletion of *Il-27p28* in the LysMCre strain was verified and is shown in **Figure S3**.

**Figure 4:**
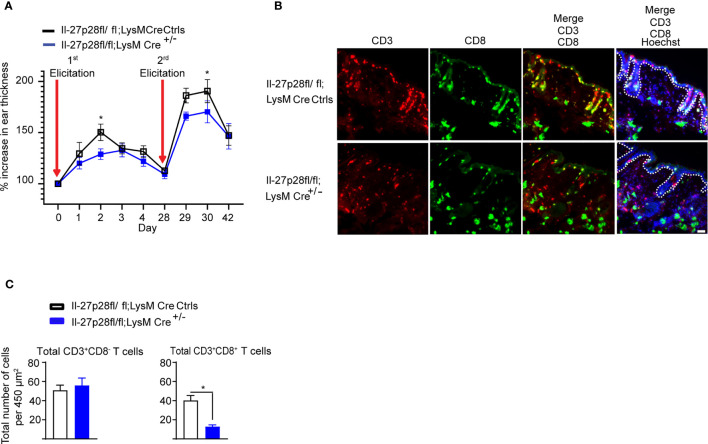
MAC-derived IL-27p28 mediates CHS inflammation and maintenance of dermal T cell numbers. **(A)** Il-27p28fl/fl;LysMCre^+/-^ and Il-27p28fl/fl;LysMCre^-/-^ control mice (Ctrls) were sensitized and elicited with DNFB 2 times. Ear swelling was measured daily and is depicted as mean ± SEM from at least 5 mice per group, *p < 0.05 (ANOVA test followed by least-significant differences multi-comparison (LSD) test). **(B)** Representative immunofluorescence staining showing CD3 (red), CD8 (green), and Hoechst (blue) in DNFB-treated back skin from Il-27p28fl/fl;LysMCre^+/-^ and Il-27p28fl/fl;LysMCre control mice (Ctrls). Data are representative of 3 mice per group. Original magnification x200 with scale bars 50 µm. White dashed lines mark the epidermal-dermal junction. **(C)** Analysis of total numbers of CD3^+^CD8^-^ and CD3^+^CD8^+^ T cells of mouse back skin. Data depicted as mean ± SEM from 3 mice per group using at least 3 microscopic views (area = 450 µm^2^), unpaired Student’s t test, *p < 0.05.

A recent study demonstrated that the magnitude of the CHS reaction, as measured by the increase in ear thickness, is strongly correlated with the number of DNFB exposures and the frequency of CD8^+^ skin T cells (4). Because dermal leukocyte clusters and CD8^+^ T cell infiltration into the skin are of transient nature in mice undergoing CHS, we adapted the ‘classical’ CHS mouse model through repeated applications of DNFB, which led to good identification and visualization of dermal and epidermal T cells, including CD8^+^ T cells. We found a consistently significant difference in the ear thickness between Il-27p28fl/fl;LysMCre and control mice (**Figure 4A**), indicating that IL-27 produced by LysM-expressing cells is indeed important for ear swelling in this CHS model. We next determined whether IL-27 from LysM^+^ cells was relevant to the skin T cell numbers (**Figures 4B, C**). Notably, Il-27p28fl/fl;LysMCre mice showed a significant reduction in total CD8^+^ T cells, but not CD3^+^CD8^-^ T cells (**Figures 4B, C**). These results indicate that IL-27 in myeloid cells is highly relevant for CD8^+^ T cell maintenance and the CHS response.

### IL-27 Regulates IL-15 Expression in CHS in a STAT1-Dependent but IFNAR1-Independent Manner

**Figure 5:**
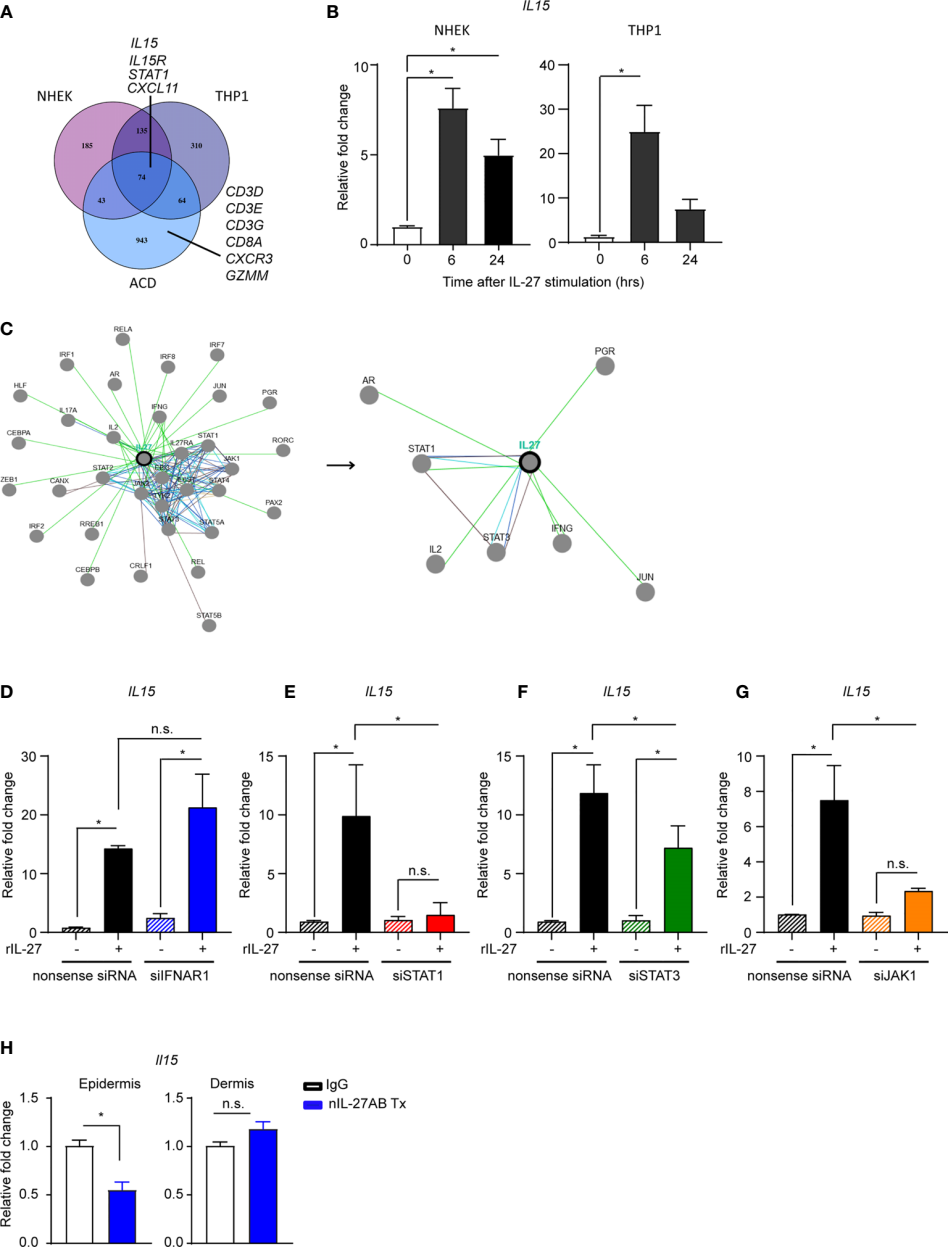
IL-27 upregulates IL-15 production through a STAT1-dependent signaling pathway. **(A)** Overlap of genes that had a p-value ≤ 0.05 and at least a 50% increase in expression in each of the three datasets gene expression datasets. For the NHEK and THP-1 datasets, the 50% increase in expression was in the recombinant human IL-27 stimulated cells (rhIL-27, 100ng/ml). **(B)** Quantitative PCR of IL15 in NHEKs and THP1 stimulated with rhIL-27 (100 ng/ml). Data are summarized as mean ± SEM from at least 3 biological replicates, *p < 0.05 (ANOVA). **(C)** The biocomputational analysis from pathwaycommon® representing the association pathways/molecules of IL-27 from open public data sets. **(D–G)** Quantitative PCR of IL15 in NHEKs transfected with siRNAs specific for **(D)** IFNAR1, **(E)** STAT1, **(F)** STAT3, and **(G)** JAK1 expression and then stimulated with rhIL-27(100 ng/ml). Data are summarized as mean ± SEM from 1-2 biological replicates *p < 0.05; n.s., not significant (ANOVA). **(H)** Quantitative PCR of ll15 in the ear epidermis and dermis from the CHS murine model using DNFB hapten allergen (0.1% DNFB, applied topically every 1-2 months). The mice received nIL-27Ab (i.d.) 40 days after elicitation and the skins were harvested 6 hours later. Data are representative of at least 3 mice per group and summarized as mean ± SEM, *p < 0.05; n.s., not significant (unpaired Student’s t test).

First, we identified through biocomputational analyses differentially expressed genes (DEGs) from 3 microarrays: 1) DEGs between vehicle and IL-27-treated THP-1 cells (GSE143228) 2) DEGs from normal human epidermal keratinocytes (NHEK) treated with vehicle or IL-27 (GSE143228), and 3) DEGs from patch-test positive ACD patient samples compared to the skin at 0 hr (GSE6281; also shown in **Figure 2A**) (**Figure 5A**). We then utilized these DEGs in an overlap analysis to reveal shared gene signatures between all groups and identified *IL-15*, *IL15R*, *STAT1* as well as other genes (**Figure 5A**). Ingenuity Pathway Analysis determined that the IL-15 production pathway was one of the top potential pathways found to be upregulated in both NHEKs and THP-1 cells treated with recombinant human IL-27 (rhIL-27) as well as in human ACD samples from 96 hours after epicutaneous challenge (adjusted p-value = 0.0014, *IL15*, *STAT1*, and *IRF1*) (**Figure 5A**, and data not shown). Quantitative-PCR analysis substantiated our computational findings showing that rhIL-27 treatment indeed upregulated *IL15* mRNA expression in keratinocytes and THP-1 cells (**Figure 5B**).

We next investigated signaling events involved in IL-27-induced *IL-15* mRNA expression in human keratinocytes. Based on our computational pathway analyses (**Figure 5C**) and prior studies in our laboratory (27), we hypothesized that STAT1 was preferentially activated as part of the IL-27 signaling pathway. To test this idea, we utilized a gene silencing approach to knockdown *IFNAR1*, *STAT1*, *STAT3*, and *JAK1* in NHEK, followed by stimulation with rhIL-27 (**Figures 5D–G**). The efficacy of silencing was confirmed by RT-qPCR (**Figure S4**). We found that the gene silencing of *STAT1* (**Figure 5E**) and *JAK1* (**Figure 5G**) in NHEK significantly decreased IL-27-induced *IL-15* mRNA expression. While STAT3 is reported to be one of the key mediators of IL-27 signaling in other cell types (47, 48), silencing of *IFNAR1* (**Figure 5D**) and *STAT3* (**Figure 5F**) had minimal effects on *IL-15* expression in NHEK cells. Taken together, our results indicate that IL-27 induced *IL-15* expression in keratinocytes is *via* JAK1/STAT1 pathway.

We next tested whether IL-27 signaling regulates IL-15 following repeated epicutaneous allergen exposures in mice. After three consecutive DNFB exposures, IL-27p28 neutralizing antibody (nIL-27p28AB) and appropriate IgG controls were injected into separate areas of mouse ear skin. Compared to IgG, the nIL-27p28AB treatment resulted in significant reduction of *Il-15* mRNA in the epidermal compartment of the skin, however, we did not observe such reduction in the dermal compartment (**Figure 5H**).

### Pharmacological Inhibition of IL-27 Suppresses CHS Response

**Figure 6:**
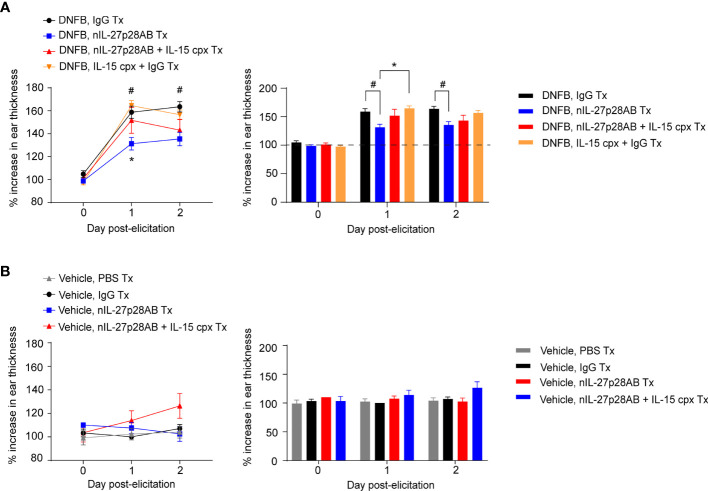
IL-15 restores skin inflammation in mice treated with neutralizing IL-27 antibody (nIL-27p28AB). **(A, B)** Wild-type (WT) mice were sensitized and elicited with **(A)** DNFB or **(B)** vehicle and treated with nIL-27p28AB, nIL-27p28AB + IL15 complex (cpx) (IL-15 + IL-15Rα), IL-15 cpx + IgG, or IgG at day 7 after the first ear elicitation. 2 days later, the mice were re-elicited with DNFB on the ear. Ear swelling was measured daily and is depicted as mean ± SEM from at least 3-4 mice per group, # and *p < 0.05 (ANOVA). # symbolizes the difference between IgG and nIL-27AB. * symbolizes the difference between nIL-27AB and IL-15 + IgG.

To strengthen the association and functional roles of IL-27 and IL-15, we next inhibited the IL-27 signaling pathway by using nIL-27p28AB in the repeated-DNFB dosing CHS model and attempted to restore the CHS response through supplementation with IL-15 complex (cpx) (IL-15 + IL-15Rα; see *Methods*). The treatment of nIL-27p28AB and/or IL-15 cpx was introduced at 7 days after the last elicitation, which is the time when IL-27p28 was preferentially upregulated in MACs following DNFB re-elicitation (**Figure 1**). Concurrent with our findings in Il-27fl/fl;LysMCre mice (**Figure 4A**), CHS ear swelling was abrogated in mice treated with nIL-27AB (**Figures 6A, B**). Complexed IL-15 (IL-15 cpx) almost completely reversed the IL-27 neutralizing effect, as shown by the increase in ear thickness with IL-15 (**Figures 6A, B**). These findings indicate that IL-27 acts through IL-15 to induce skin inflammation and ear swelling in the DNFB-induced CHS model.

### IL-15 but Not IL-27 Increases Pro-Survival BCL2 in Human Skin T Cells

**Figure 7:**
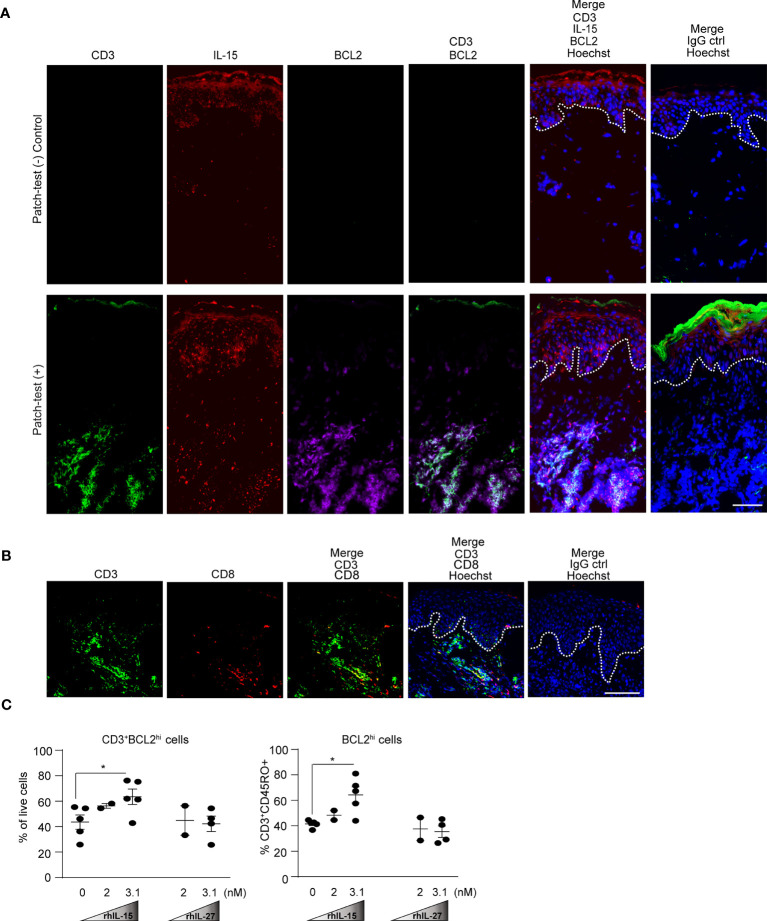
IL-15 enhances human BCL2 expression in skin T cells. **(A)** Representative immunofluorescence staining of CD3 (green), IL-15 (red), BCL2 (purple), and Hoechst (blue) in human donor-matched patch-test negative control and patch-test (+) ACD skin. Data are representative of patient samples per tested condition (at least 3 samples per condition). Original magnification x400 (stitched images) with scale bars of 100 µm. White dashed lines mark the epidermal-dermal junction. **(B)** Representative immunofluorescence staining of CD3 (green), CD8 (red), and Hoechst (blue) in human patch-test (+) ACD skin. Data are representative of patient samples per tested condition (at least 3 samples per condition). Original magnification x400 with scale bars 100 µm. White dashed lines mark the epidermal-dermal junction. **(C)** Quantification of flow cytometry analysis of healthy human skin explant T cells treated with rhIL-15 and rhIL-27 for 24 hours. Cells were gated on live cells and CD3^+^CD45RO^+^cells subsequently gated on CD3^+^ BCL2hi and BCL2hi cells, respectively. Data are summarized as mean ± SEM, *p < 0.05 (paired Student’s t test).

We observed expression of IL-15 specifically in the epidermis and the dermal leukocyte clusters of patch-test positive skin samples (**Figure 7A**). In agreement with our prior findings (29), we identified that CD14^+^iNOS^+^ myeloid cells within the leukocyte clusters produced IL-15 (**Figure S5**). IL-15 has been known to up-regulate pro-survival BCL2 in memory T cells of contact allergen-experienced mice (4, 49–53). We found that T cells within dermal leukocyte clusters, comprising of CD8^+^ and CD8^-^ T cells as well as some non-T cells expressed high BCL2 in the patch-test positive skin (**Figures 7A, B**).

We next examined the effect of IL-15 versus IL-27 on BCL2 expression in T cells using human skin explants which allowed the analysis of the characteristic skin-resident memory CD45RO^+^ T cells (32, 54). The frequency of skin T_RM_ (CD3^+^CD45RO^+^) within our experiment was consistently over 70 percent of the total sorted skin T cells (**Figure S6.1**). When treated with rhIL-15, CD45RO^+^ T cells increased their frequency and intensity of BCL2^hi^, but not BCL-XL, another important member of the BCL2 family (**Figure 7C**, **Figure S6.2**). In contrast, treating sorted T cells from skin explants with human rhIL-27 at higher or equimolar concentration to IL-15, IL-27 did not increase the BCL2 nor BCL-XL expression (**Figures 7C** and **S6.2**). These results indicate that IL-27 did not directly enhance T cell survival, but instead activated other cells to produce IL-15 which then stimulated pro-survival signals in T cells.

### IL-27 and IL-15 Are Required for T Cell Maintenance After the Resolution of DNFB-Mediated Skin Inflammation

**Figure 8:**
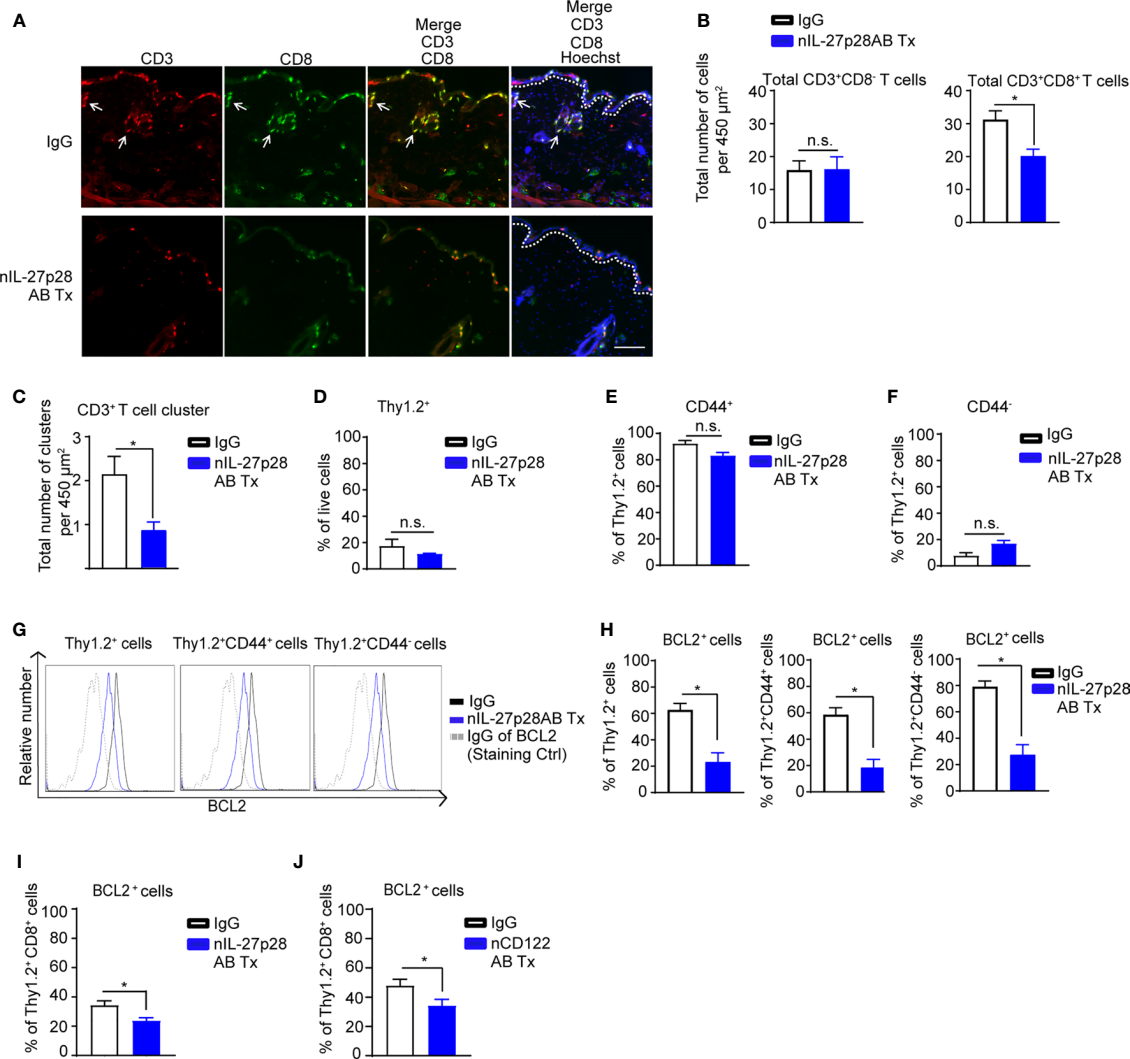
IL-27p28 is required for T cell maintenance post-elicitation. **(A)** Representative immunofluorescence staining showing CD3 (red), CD8 (green), and Hoechst (blue) in back skin from the CHS mice treated with neutralizing IL-27p28 antibody (nIL-27p28AB) or control IgG. The mice started to receive nIL-27p28AB 70 days after elicitation and the mouse skin were harvested 2 days after the first treatment. White arrows indicate leukocyte clusters. Data are representative of 4 mice. Original magnification x200 with scale bars 100 µm. White dashed lines mark the epidermal-dermal junction. **(B)** Analysis of total numbers of CD3+CD8- and CD3+CD8+ T cells of mouse back skin following nIL-27p28AB treatment. Data are depicted as mean ± SEM from 4 mice per group using at least 3 microscopic views (area = 450 µm^2^), *p < 0.05; n.s., not significant (unpaired Student’s t test). **(C)** Analysis depicting total numbers of CD3^+^ T cell clusters (size of CD3^+^ cell diameter > 20 µM) of mice back skin upon treatment with nIL-27p28AB (6 µg, 3 times twice a day). Data are depicted as mean ± SEM from 4 mice per group using at least 3 microscopic views (area = 450 µm^2^), *p < 0.05 (unpaired Student’s t test). **(D–H)** Flow cytometry analysis of repetitive DNFB exposure mouse ear upon injection with nIL-27p28AB (12 µg) or IgG vehicle control for 2 days showing mean frequency ± SEM of total **(D)** Thy1.2^+^ cell and **(E)** CD44^+^ and **(F)** CD44^-^ cells in Thy1.2^+^ cells. Representative **(G)** histogram and **(H)** frequency of BCL2 that are gated on Thy1.2^+^, Thy1.2+CD44^+^, or Thy1.2^+^CD44^-^ cells. Data are depicted as mean plusmn; SEM from at least 4 mice per group, *p < 0.05; n.s., not significant (unpaired Student’s t test). **(I)** Same experimental setting as in **(D)**, the mice either received nIL-27p28AB or their appropriate control IgG. Flow cytometry analysis depicting the mean frequency ± SEM of total BCL2^+^ in Thy1.2^+^CD8^+^ cells for at least 4 mice per group and summarized as mean ± SEM, unpaired Student’s t test, *p < 0.05. **(J)** Similar experimental setting as in **(I)**, the mice either received neutralizing CD122 antibody (nCD122AB) or their appropriate control IgG. Flow cytometry analysis depicting the mean frequency ± SEM of total BCL2^+^ in Thy1.2^+^CD8^+^ cells from at least 4 mice per group and summarized as mean ± SEM, unpaired Student’s t test, *p < 0.05.

Our results support a role of IL-27 at both elicitation and resolution of inflammation. During the resolution phase, effector T cells either undergo apoptosis or differentiate into memory T cells (55, 56). Skin T_RM_ cells are maintained through IL-7 and IL-15 production by keratinocytes (6). In our experimental setting, IL-7 was not regulated through IL-27 (data not shown). Therefore, we next examined the effect of our identified IL-27-IL-15 signaling axis on T cell survival in allergen-experienced mice using nIL-27p28AB or neutralizing CD122 antibody (nCD122AB, IL-15 signaling blocking antibody) (57). Following repeated DNFB exposures, we injected nIL-27p28AB or appropriate IgG controls into separate areas of previously DNFB-exposed mouse back skin and harvested skin for analysis. Immunofluorescence staining and quantitative analyses revealed a significant reduction of total CD8^+^ T cells, but not CD3^+^CD8^-^ T cells, upon nIL-27p28AB treatment (**Figures 8A, B**). The number of CD3^+^ T cell clusters significantly decreased in the back skin of the mice treated with nIL-27p28AB compared to the IgG-treated back skin (**Figure 8C**).

We next tested the effect of IL-27p28 blockade on T cell survival of DNFB-experienced mice using flow cytometry. Administration of nIL-27p28AB did not alter the overall frequency of Thy1.2^+^ cells, Thy1.2^+^CD44^+^, or Thy1.2^+^CD44^-^ T cells (**Figures 8D–F**; **Figure S7**). However, the nIL-27p28AB treatment resulted in reduction of BCL2 frequency and fluorescence intensity in Thy1.2^+^CD44^+^ and Thy1.2^+^CD44^-^ cells compared to the IgG control group (**Figures 8G, H**; **Figure S7**).

In DNFB-experienced mice treated with nIL-27p28AB or nCD122AB, the frequency of BCL2^+^ cells within skin CD8^+^ T cell population significantly decreased compared to that of the IgG control group (**Figures 8I, J**; **Figures S7** and **S8**). These data demonstrate that the IL-27/IL-15 signaling axis is required for the long-term maintenance for a subset of CD8^+^ T cells after skin exposure to allergens.

## Discussion

Here, we report that IL-27 activates both epithelial keratinocytes and myeloid cells within dermal leukocyte clusters to produce the key T cell ‘survival cytokine’ IL-15 following epicutaneous allergen exposure. We identified upregulated IL-27 expression in skin of patch-test positive patient skin samples and in CD14^+^CD86^+^ human monocyte/MACs. In agreement with the clinical presentation of delayed-type IV hypersensitivity reactions typically occurring within 72-96 hours post-patch testing (58), we found these IL-27-producing myeloid cells are recruited to dermal leukocyte clusters in the skin at 96-120 hours post-allergen exposure. Notably, we identified that hapten stimulation of human monocytic THP-1 cells *in vitro* leads to the induction of IL-27p28. Furthermore, CHS mouse modeling revealed that the expanded CD172a^+^ MACs population following *in vivo* hapten exposure also expressed upregulated IL-27p28. Our studies showed that nIL-27p28AB treatment in mice limited epidermal IL-15 production, dermal CD8+ T cell numbers, and pro-survival BCL2 expression in T cells. This finding, however, does not exclude the possibility that the reduced T cell number is linked to decreased cell proliferation and/or skin infiltration. Moreover, the functional *in vivo* studies confirmed this connection by demonstrating that the CHS suppression through nIL-27p28AB was abrogated when IL-15 cpx (IL-15 + IL-15Rα) was co-administered. Mechanistically, IL-27 acts through induction of IL-15 to promote T cell survival in CHS skin. Our studies unveiled crucial roles of IL-27 and IL-15 in cutaneous allergic immunity.

Given the complexity of the IL-27 signaling regulatory system, it is not surprising that IL-27 signaling is implicated in both pro- and anti-inflammatory settings (10, 13, 16–18, 21–26, 48, 59–68). IL-27 consists of IL-27p28 and EBI3; EBI3 can also heterodimerize with p35 to form the ‘regulatory’ cytokine IL-35 (63). IL-27 signals through a receptor complex consisting of IL27RA and GP130 (63). GP130 is also known to heterodimerize with other receptor subunits to form receptor complexes to facilitate signaling for IL-6, IL-11, IL-35, leukemia inhibitory factor (LIF) and many others (69). The availability of heterodimerization partners of both the ligands and the receptors ultimately determine the outcomes of IL-27 signaling. In this regard, it has been previously reported that mice with a global deletion of *Ebi3* or *Il27ra* showed increased delayed-type hypersensitivity responses (17, 23). *Ebi3^-/-^* mice had increased delayed-type hypersensitivity responses, but the significant effect within the ear swelling kinetics is rather late, peaking at around 48 hours and not at the peak inflammation time point, around 24 hours (23). In addition, mice lacking *Ebi3* or *Il27ra* may activate yet unknown compensatory mechanisms, which could lead, for example, to a shift in cytokine production or responsiveness in various cells. The global knockout may also variably affect myeloid and/or T cell development and availability. Future studies are needed to address these effects in further detail.

Upregulated IL-27 production was found in MACs at 7 days after DNFB elicitation, a time point when resolution of allergic skin inflammation begins. Although various IL-27-activating stimuli and conditions have been reported (25, 63), we do not know with certainty what pathway leads to IL-27 upregulation following epicutaneous allergen exposures. While we reported that experimental skin wounding induces IL-27p28 production by CD301b^+^ monocyte-derived DCs and MACs (25), during vaccine-elicited cellular immunity, XCR1^+^ DCs and monocytes were identified as key IL-27p28 producers (14, 15). In our current study, we showed that CD172^+^ MACs produce IL-27p28 whereas CD45^+^Lin^-^CD11b^-^CD24^-/lo^ cells, mainly comprising of XCR1^+^CD172a^-^ DCs, demonstrate no change in IL-27p28 production following *in vivo* allergen exposure.

F4/80 and CD68 can be expressed by both MACs as well as dermal DCs and are not the most reliable maker for MACs in the skin (42, 70). Therefore, we chose an in-depth skin immune cell identification in this study according to Tamoutounour et al. (42). Interestingly, we found that approximately 50% of IL-27-expressing MACs population (CD45^+^Lin^-^CD11b^+^CD24^-/lo^ CD64^hi^CCR2^-/lo^) of the DNFB treated ears expressed F4/80 and 40% of them expressed CD86. However, CD86 is expressed by both MACs and Langerhans cells in human and mouse skin (71, 72). For this reason, we used immunofluorescent staining to locate CD86^+^ MACs in the dermal compartment of human skin. In our assays, the number of CD11b^+^ DCs that express IL-27 following DNFB challenge is rather limited. In addition, less than 10% of IL-27-expressing CD11b^+^ DCs (CD45^+^Lin^-^CD11b^+^CD24^-/lo^CD64^-^Ly-6C^-^) expressed CD11c, which could be due to enzymatic digestion during tissue processing (73). It is also possible that, upon hapten exposure, mouse DCs down-regulate CD11c through toll-like receptors 3, 4, and/or 9 which are activated and contribute to CHS (74–77).

We utilized Il-27p28fl/fl;LysMCre mice to investigate the role of IL-27-producing MACs in CHS immunity. LysMCre can target both MACs and neutrophils, but not DCs. Since neutrophilic inflammation in CHS is typically observed early on and is rather a transient response, we believe that the potential of IL-27 production by neutrophils may not impair the interpretation of our studies as we focus on the phase between elicitation and resolution of skin inflammation (78, 79). Furthermore, a recent study using an anti-Ly6G (1A8) in CHS studies found no correlation between neutrophils and DCs-MACs-T cell clusters (28). We showed that compared to controls, Il-27p28fl/fl;LysMCre mice have significantly mitigated CHS inflammatory responses *in vivo*, identifying an important role of IL-27p28 in CHS immunity. Intriguingly, upon repeated DNFB exposures, neither *Il-27p28* conditional knockout mice nor mice treated with the IL-27p28 neutralizing antibody demonstrated a complete abolishment of CHS-induced ear swelling, indicating that additional factors are at play.

We found IL-27 to be predominantly expressed by human CD14^+^CD86^+^CD172a^+^ MACs at 96-120 hours after patch-testing. CD172a, also called SIRPα, interacts with signal regulatory protein CD47, with known roles in eliciting “do not eat me” signals (44–46). Dysregulation of the CD172a-CD47 interaction has been associated with various diseases (44–46). Indeed, we observed high CD47 expression on T cells within dermal leukocyte clusters, which were located in immediate proximity to CD172a^+^CD14^+^ cells in our skin biopsy samples. While our current work focused on IL-27 function in MACs, the functional interaction and role of CD172a and CD47 in CHS is an exciting avenue for future research as well.

Tissue-resident memory T cells (T_RM_) is essential for eliciting a rapid and robust CHS response following hapten re-exposure (80). Central memory T cells (T_CM_) can differentiate to T_RM_, and therefore also contribute to CHS severity (80). These adaptive immune activities are regulated by innate immune cells (28, 29). The number of the dermal leukocyte clusters comprising DCs, MACs, and T_RM_ cells is associated with the severity of skin inflammation, and deletion of DCs and MACs abrogated skin inflammatory response (28, 29). In contrast, deletion of Langerhans cells in the epidermis did not reduce inflammation (28, 81, 82), suggesting that dermal DCs and MACs play dominant roles in CHS. We found that MAC-derived IL-27 signaling through IL-15 plays a significant role in maintaining resident CD8^+^ T cell population in hapten-induced CHS. This, however, does not exclude the potential effect of IL-15 and/or IL-27 derived from other sources such as the local lymph nodes. In this regard, CD68^+^ MACs, but not DCs, produce IL-27 in human lymph nodes (83). In addition, IL-15 is produced in T-cell zone and medulla in lymph node by non-immune cells such as blood endothelial cells and fibroblastic reticular cells (84). Thus, delineating the role of local lymph node-derived IL-27 and IL-15 in CHS may be an area of future research.

In conclusion, our findings report novel roles for IL-27 and IL-15 in allergic contact sensitivity of the skin. Our work provides new insight into the immunobiology of CHS and the identification of novel targets for development for treatment regimens that may be directed to alleviating cutaneous allergic hypersensitivity through T cell modification.

## Data Availability Statement

The original contributions presented in the study are included in the article/**Supplementary Material**. Further inquiries can be directed to the corresponding author.

## Ethics Statement

The studies involving human participants were reviewed and approved by Institutional Review Board of Duke University Health System. The patients/participants provided their written informed consent to participate in this study. The animal study was reviewed and approved by Duke University Institutional Animal Care and Use Committee.

## Author Contributions 

JS, ML, PH, JK, JZ, and AM wrote the paper; all authors reviewed and edited the paper. JS, ML, JZ, and AM planned experiments. JS, ML, PH, JK, and LF performed experiments. JS, ML, PH, JK, JZ, and AM analyzed experiments. JSS, AA, and SR wrote IRB protocol, collected, and provided human skin samples. DC analyzed all microarray and Venn diagram data. RK generated and provided Il-27p28EGFP mice. ZY generated and provided Il-27fl/fl mice. All authors contributed to the article and approved the submitted version.

## Funding

This work is supported by a National Institutes of Health R01AI139207 to AM and JZ.

## Conflict of Interest

Author AM has received funding from Silab, Inc. and has consulted for the company. AM also served on the reviewer committee for the LEO foundation and the Triangle Community Foundation. Author AA received a Pfizer Independent Grant for Learning and Change and has consulted for Henkel.

The remaining authors declare that the research was conducted in the absence of any commercial or financial relationships that could be construed as a potential conflict of interest.

## Publisher’s Note

All claims expressed in this article are solely those of the authors and do not necessarily represent those of their affiliated organizations, or those of the publisher, the editors and the reviewers. Any product that may be evaluated in this article, or claim that may be made by its manufacturer, is not guaranteed or endorsed by the publisher.
