# Supplementary material for: IL-27 Derived From Macrophages Facilitates IL-15 Production and T Cell Maintenance Following Allergic Hypersensitivity Responses
Source: Front Immunol. 2021 Sep 30;12:713304. doi: 10.3389/fimmu.2021.713304 (PMC8515907; doi:10.3389/fimmu.2021.713304)
Supplement: Supplementary file 1 [file DataSheet_1.pdf]

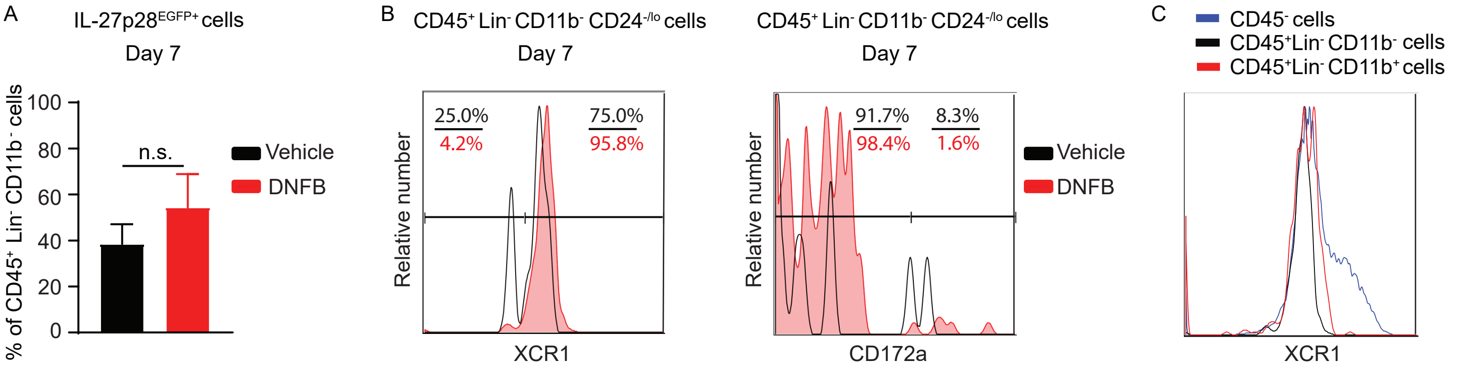

Figure S1. Allergen exposure in mouse ears
(A) Quantitative analysis of Il-27p28^{EGFP+} cells (in CD45^{+}Lin^{-} CD11b^{-} CD24$^{-/lo}$ cells) in DNFB elicited ears in back sensitized mice, n.s., not significant (unpaired Student's t test).
(B) Representative staining for XCR1 and CD172a for flow cytometry analysis.
(C) Histograms from representative flow cytometry analysis for XCR1 of DNFB-treated ears (7 days post-elicitation).

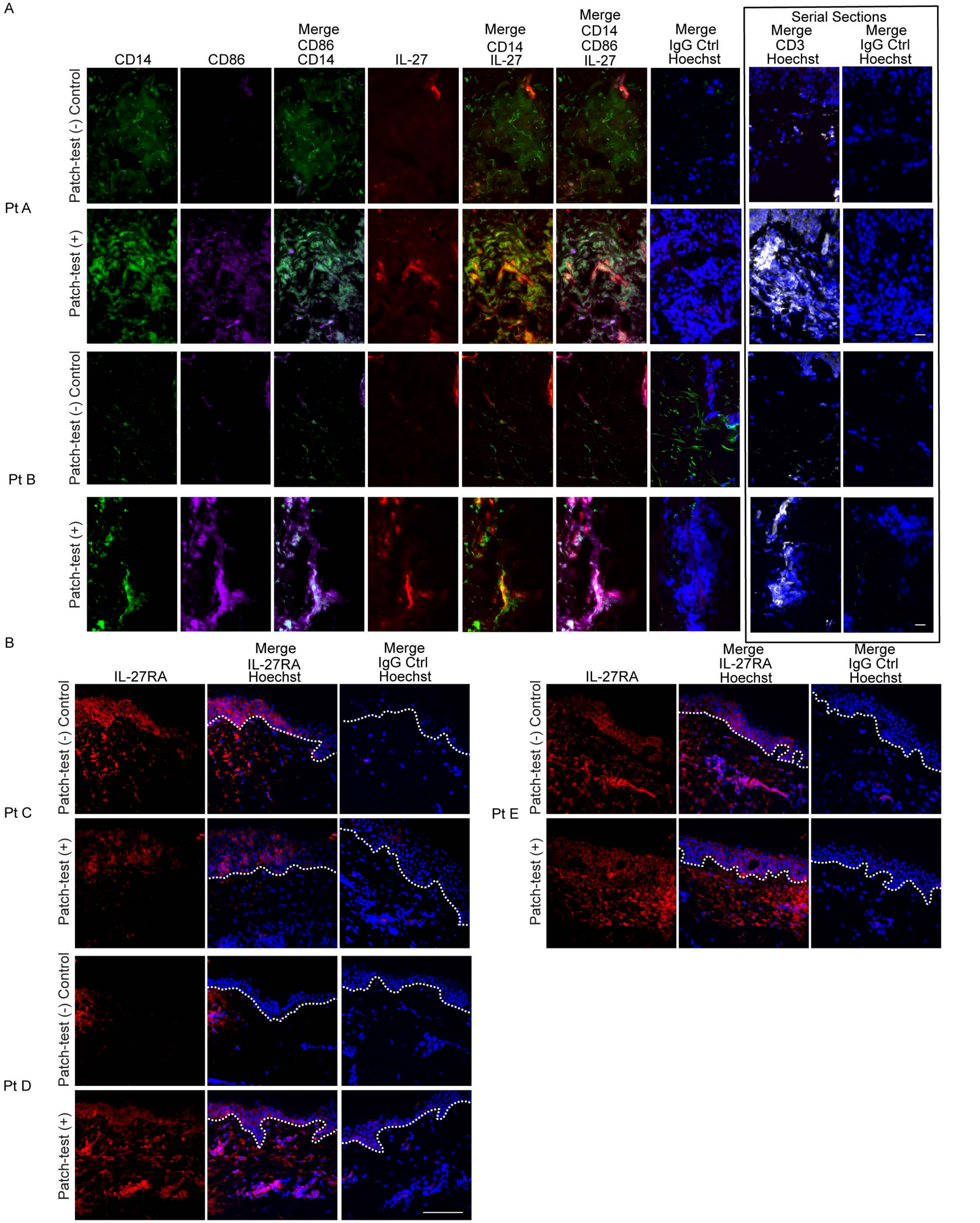

Figure S2. Exposure to allergen induces IL-27 in human with ACD

(A) Representative immunofluorescence staining of IL-27 (red), CD14 (green), CD86 (purple), CD3 (white) and Hoechst (blue) in human donor-matched patch-test negative control and patch-test (+) ACD skin. Data are representative of patient samples A and B per stained condition. Original magnification x400 with scale bars 20 μm. (Pt is an abbreviation for patient)

(B) Representative immunofluorescence staining of IL-27RA (red) and Hoechst (blue) in human donor-matched patch-test negative control and patch-test (+) ACD skin. Data are representative of patient samples C, D, and E per stained condition. Original magnification x400 with scale bars 100 μm.White dashed lines mark the the epidermal-dermal junction. (Pt is an abbreviation for patient)

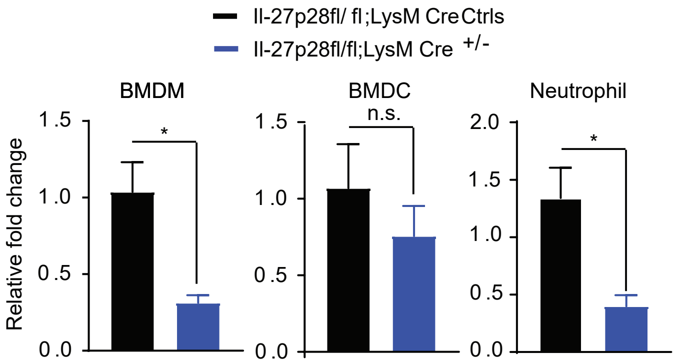

Figure S3. Validation of the Il-27p28fl/fl;LysM Cre
Quantitiative PCR of *Il-27p28* of bone marrow–derived MACs (BMDM), bone marrow–derived DCs (BMDC) and sorted-neutrophils from the bone marrow from Il-27p28fl/fl;LysM Cre mice and their controls (Ctrl). Data are summarized as mean ± SEM from at least 3 mice per group, *p < 0.05; n.s., not significant (unpaired Student's t test).

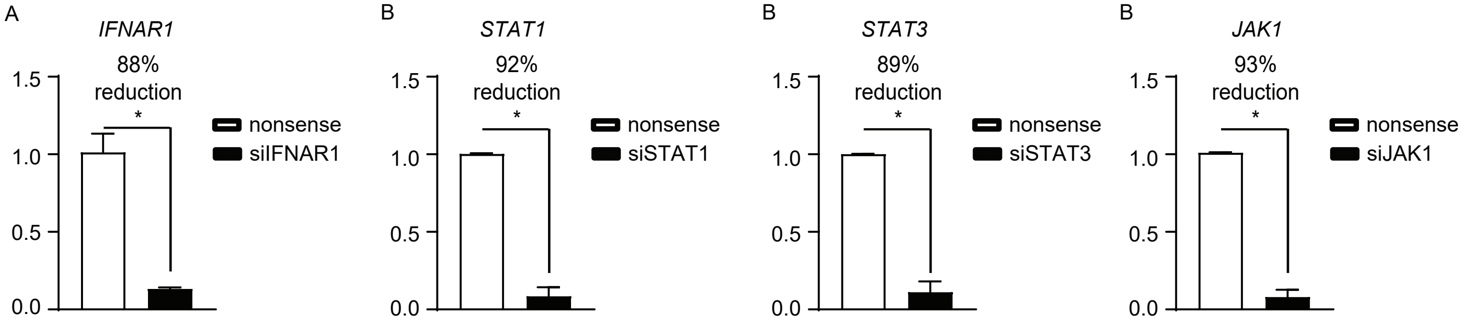

Figure S4. *IFNAR1*, *STAT1*, *STAT3* and *JAK1* silencing by siRNA
(A) *IFNAR1* gene expression in NHEK as compared to nonsense ctrl, *p < 0.05 (n=1, unpaired Student's t test).
(B) *STAT1* gene expression in NHEK as compared to nonsense ctrl, *p < 0.05 (n=2, paired Student's t test).
(C) *STAT3* gene expression in NHEK as compared to nonsense ctrl, *p < 0.05 (n=2, paired Student's t test).
(D) *JAK1* gene expression in NHEK as compared to nonsense ctrl, *p < 0.05 (n=2, paired Student's t test).

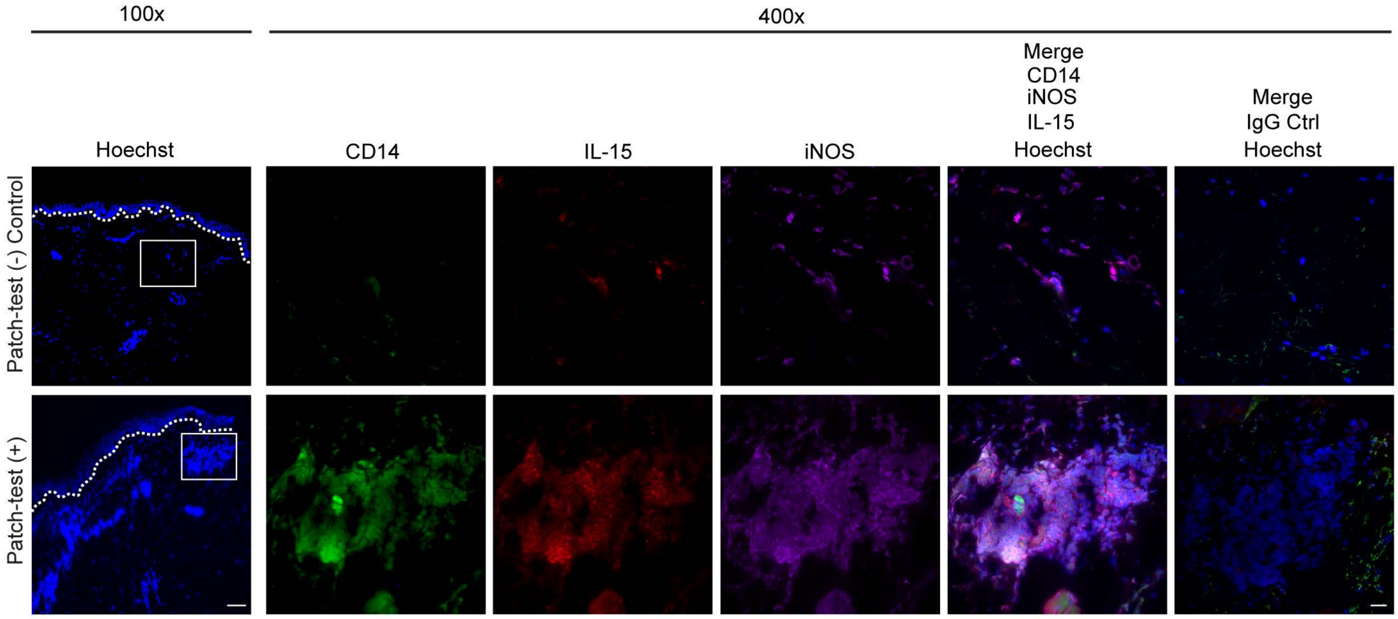

Figure S5. Increased IL-15 expressing CD14^{+}iNOS^{+} myeloid cells within leukocyte clusters in ACD skin

Representative immunofluorescence staining of CD14 (green), IL-15 (red), iNOS (purple) and Hoechst (blue) in human donor-matched patch-test negative control and patch-test (+) ACD skin. Data are representative of patient samples (at least n=3) per stained condition. Original magnification x100 (left) and original magnification x400 (right) with scale bars 100 µm, and 20 µm, respectively. White dashed lines mark the epidermal-dermal junction.

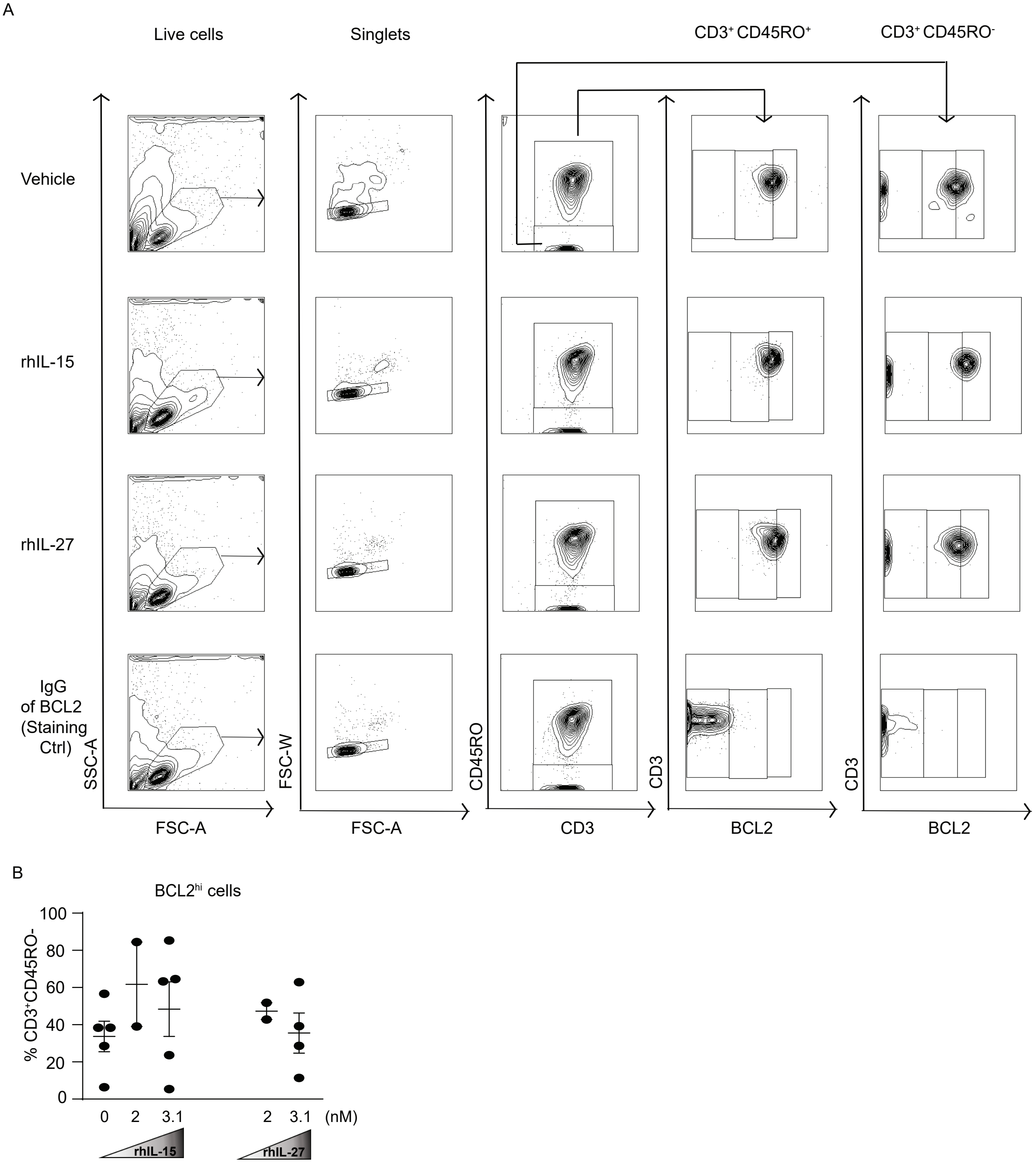

Figure S6.1. IL-15 enhances BCL2 expression in human T_{RM}
(A) Gating strategy related to Figure 7.
(B) Quantitative analysis depicting the mean frequency ± SEM of total BCL2^{hi} cells in CD3^{+}CD45RO^{-}cells from human skin explant T cells treated with rhIL-15 and rhIL-27 for 24 hrs. (paired Student’s t test).

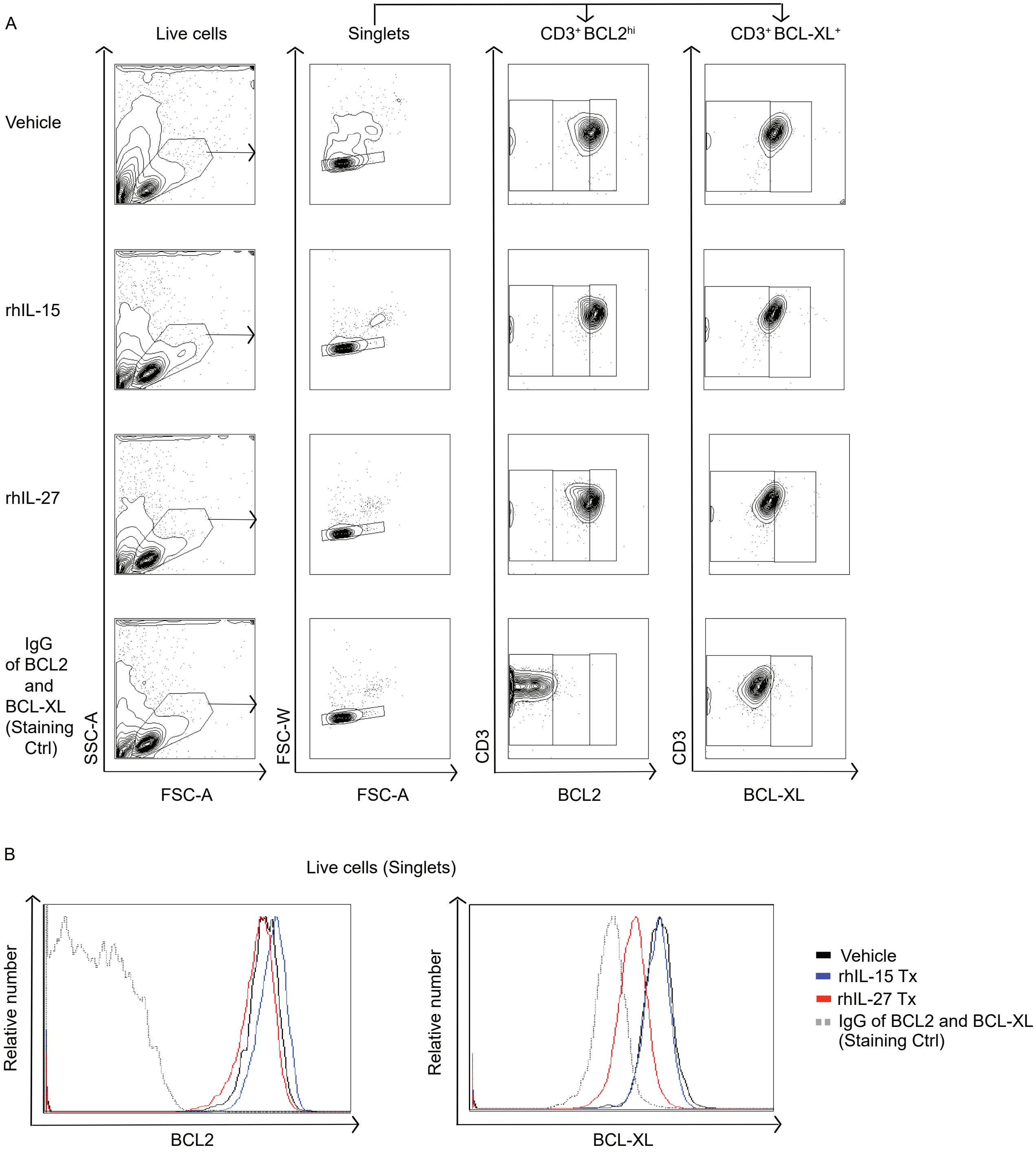

Figure S6.2. IL-15 enhances BCL2 expression in human resident T cells
(A) Gating strategy related to Figure 7.
(B) Histograms from representative flow cytometry analysis for BCL2 and BCl-XL of human skin explant T cells treated with rhIL-15 and rhIL-27 for 24 hrs.

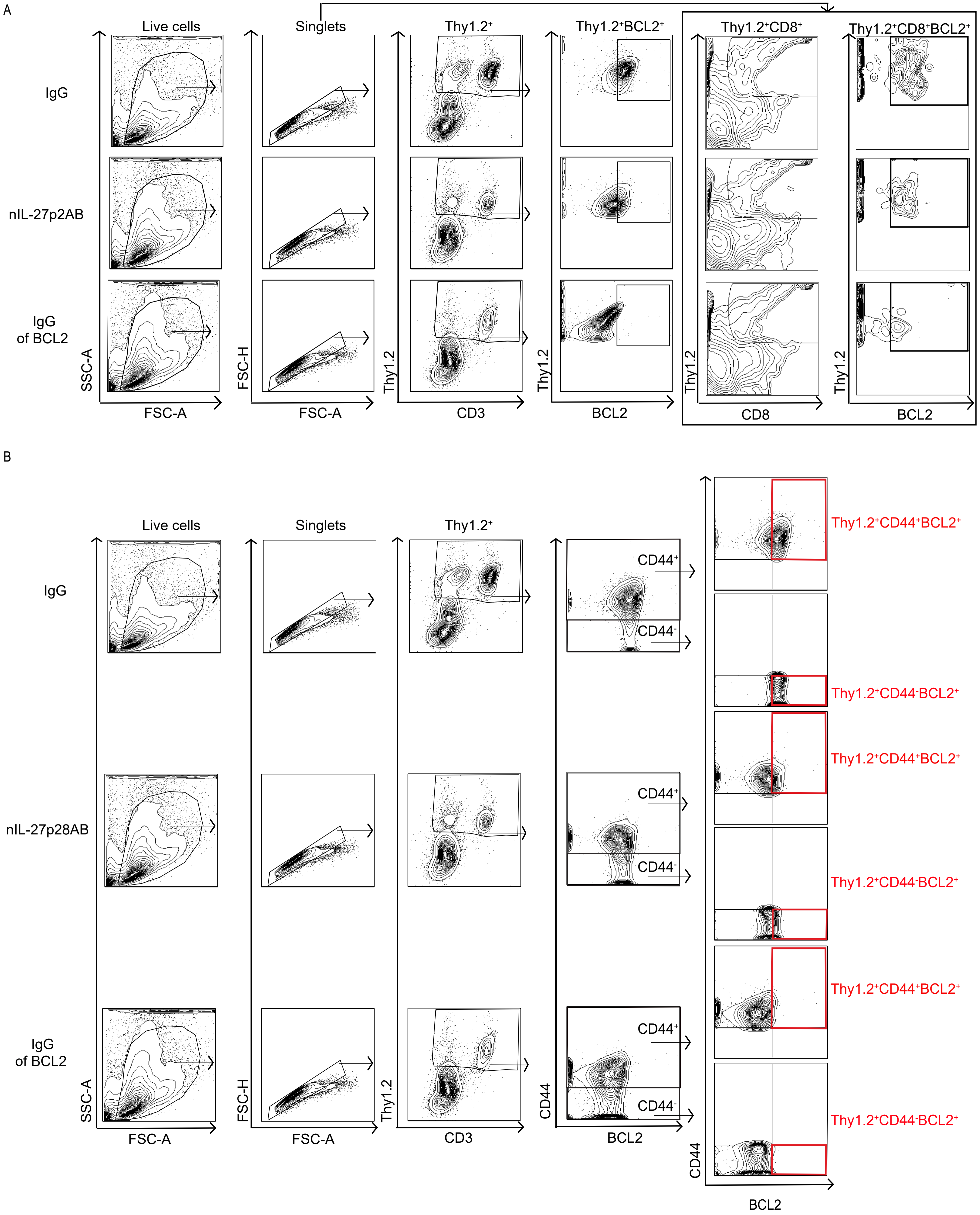

Figure S7. Gating strategy related to Figure 8D-I

A

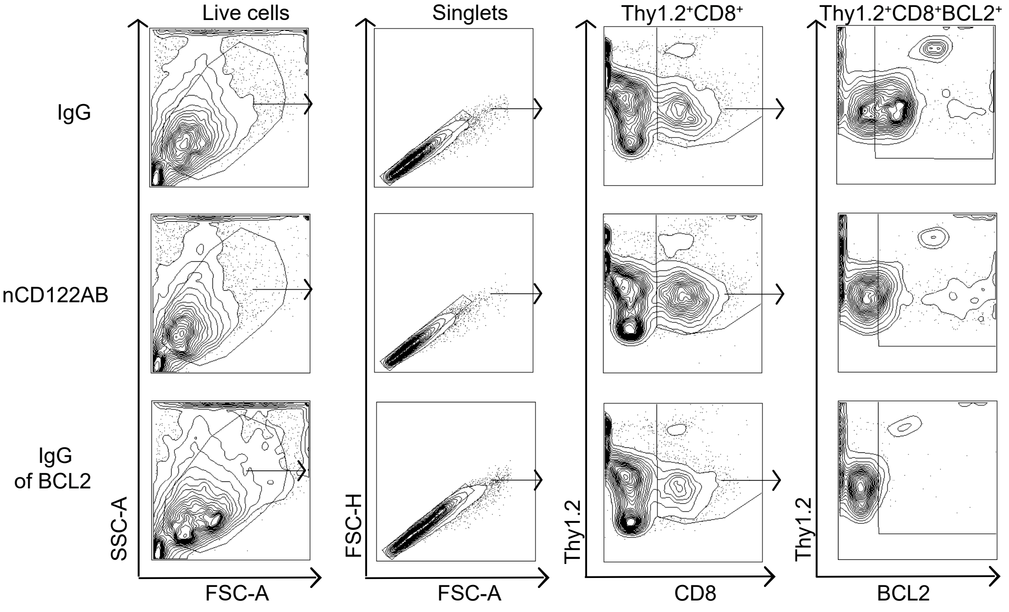

Figure S8. Gating strategy related to Figure 8J
