# Supplementary material for: IL-27 Derived From Macrophages Facilitates IL-15 Production and T Cell Maintenance Following Allergic Hypersensitivity Responses
Source: Front Immunol. 2021 Sep 30;12:713304. doi: 10.3389/fimmu.2021.713304 (PMC8515907; doi:10.3389/fimmu.2021.713304)
Supplement: Supplementary file 2 [file Table_1.docx]

Supplementary materials for

**IL-27 derived from macrophages facilitates IL-15 production and T cell maintenance following allergic hypersensitivity responses**

Jutamas Suwanpradid, Min Jin Lee_,_ Peter Hoang, Jeffery Kwock, Lauren P. Floyd, Jeffrey S. Smith, Zhinan Yin, Amber R. Atwater, Sudarshan Rajagopal, Ross M. Kedl, David L. Corcoran, Jennifer Y. Zhang*, Amanda S. MacLeod*

*Corresponding author email:

[amanda.macleod@duke.edu](mailto:amanda.macleod@duke.edu);

[jennifer.zhang@duke.edu](mailto:jennifer.zhang@duke.edu)

Number of supplementary tables:

1

Number of supplementary figures:

9

**Table 1:** **Key oligonucleotides for RT-PCR and siRNA experiments**

| **Oligonucleotides** | **SOURCE** | **IDENTIFIER** |
| --- | --- | --- |
| Human *GAPDH* forward primer:  5'-ATGGGAAGGTGAAGGTCGGA-3'  Human *GAPDH* reverse primer:  5'-CAGCGTCAAAGGTGGAGGAGT-3' | Yang et al.^1^ | N/A |
| Human *IL27p28* forward primer:  5'-GAGCAGCTCCCTGATGTTTC-3'  Human *IL27p28* reverse primer:  5'-AGCTGCATCCTCTCCATGTT-3' | Anuradha et al.^2^ | N/A |
| Human *IL15* forward primer:  5'-TTTGGGCTGTTTCAGTGCAG-3'  Human *IL15* reverse primer:  5'-ACTTTGCAACTGGGGTGAAC-3' | This study | N/A |
| Mouse *Gapdh* forward primer:  5'-AGGTCGGTGTGAACGGATTTG-3'  Mouse *Gapdh* reverse primer:  5'-TGTAGACCATGTAGTTGAGGTCA-3' | Cool et al.^3^ | N/A |
| Mouse *Il15* forward primer:  5'-GGCATTCATGTCTTCATTTTGG-3'  Mouse *Il15* reverse primer:  5'-TCCAGTTGGCCTCTGTTTTAGG-3' | Chin et al.^4^, Yim et al.^5^ | N/A |
| Mouse *Il27p28* forward primer:  5'-CTGAATCTCGATTGCCAGGAGTGA-3'  Mouse *Il27p28* reverse primer:  5'-AGCGAGGAAGCAGAGTCTCTCAGAG-3' | Wei et al. ^6^ | N/A |
| [*IFNAR1* Human siRNA Oligo Duplex (Locus ID 3454)](https://www.origene.com/catalog/rnai/sirna-oligo-duplexes/sr302334/ifnar1-human-sirna-oligo-duplex-locus-id-3454) | OriGene  Technologies | Cat# SR302334 |
| [*STAT1* Human siRNA Oligo Duplex (Locus ID 6772)](https://www.origene.com/catalog/rnai/sirna-oligo-duplexes/sr321905/stat1-human-sirna-oligo-duplex-locus-id-6772) | OriGene Technologies | Cat# SR304620 |
| [*STAT3* Human siRNA Oligo Duplex (Locus ID 6774)](https://www.origene.com/catalog/rnai/sirna-oligo-duplexes/sr321907/stat3-human-sirna-oligo-duplex-locus-id-6774) | OriGene Technologies | Cat# SR304622 |
| *JAK1* Human siRNA Duplex (Locus ID 3716)  5'- CCACAUAGCUGAUCUGAAA-3'  5'- UGAAAUCACUCACAUUGUA-3'  5'- UAAGGAACCUCUAUCAUGA-3' | Dharmacon | Cat#  D-003145-05  D-003145-06  D-003145-07 |
| [Scrambled negative control siRNA duplex](https://www.origene.com/catalog/rnai/sirna-oligo-duplexes/sr30004/sirna-related-product) | OriGene Technologies | Cat# SR30004 |

**Reference:**

1 Yang B, Suwanpradid J, Sanchez-Lagunes R, Choi HW, Hoang P, Wang D, et al. IL-27 Facilitates Skin Wound Healing Through Induction of Epidermal Proliferation and Host Defense. *J Invest Dermatol* (2017) 137:1166–75. doi: 10.1016/j.jid.2017.01.010.

2 Anuradha R, Munisankar S, Bhootra Y, Dolla C, Kumaran P, Nutman TB, et al. Modulation of CD4(+) and CD8(+) T Cell Function and Cytokine Responses in Strongyloides Stercoralis Infection by Interleukin-27 (IL-27) and IL-37. *Infect Immun* (2017) 85:e00500-17. doi: 10.1128/IAI.00500-17

3 Cool J, DeFalco TJ, Capel B. Vascular-mesenchymal cross-talk through Vegf and Pdgf drives organ patterning. *Proc Natl Acad Sci U S A* (2011) 108:167-72. doi:10.1073/pnas.1010299108

4 Chin AI, Miyahira AK, Covarrubias A, Teague J, Guo B, Dempsey PW, et al*.* Toll-like receptor 3-mediated suppression of TRAMP prostate cancer shows the critical role of type I interferons in tumor immune surveillance. *Cancer Res* (2010) 70:2595-603. doi:10.1158/0008-5472.CAN-09-1162.

5 Yim HY, Park C, Lee YD, Arimoto K, Jeon R, Baek SH, et al. Elevated Response to Type I IFN Enhances RANKL-Mediated Osteoclastogenesis in Usp18-Knockout Mice. *J Immunol* (2016) 196:3887-95. doi:10.4049/jimmunol.1501496

6 Wei J, Xia S, Sun H, Zhang S, Wang J, Zhao H, et al. Critical role of dendritic cell-derived IL-27 in antitumor immunity through regulating the recruitment and activation of NK and NKT cells. *J Immunol* (2013) 191:500-08. doi:10.4049/jimmunol.1300328
